# Home Health Nursing Care Time for Patients with Parkinson’s Disease

**DOI:** 10.3390/jpm12050714

**Published:** 2022-04-29

**Authors:** Yumi Iwasa, Miyoko Suzuki, Izumi Saito

**Affiliations:** 1 Department of Nursing, Morinomiya University of Medical Sciences, 1-26-16 Nanko-kita, Suminoe-ku, Osaka 558-8611, Osaka, Japan; 2 Department of Nursing, Graduate School of Health Sciences, Kobe University, 7-10-2 Tomogaoka, Suma-ku, Kobe 654-0142, Hyogo, Japan; izumi-saito@kitty.kobe-u.ac.jp; 3 Akebi, Home-Visit Nursing Station, 836-A Anase-Ueki, Shikama-ku, Himeji 672-8030, Hyogo, Japan; houkan@akebi.or.jp

**Keywords:** home health nursing, Parkinson’s disease, time and motion studies, time management

## Abstract

There is an urgent need to provide personalized care more efficiently to patients with Parkinson’s disease (PD) who live at home. To understand the impact of patient attributes and features on nursing care time, we assessed the amount of time required to perform home visits to patients with PD and identified patient characteristics related to differences in nursing care time. Twenty patient (median age 78.0 years) visits were video-recorded. Nursing care activities were categorized, while time spent on them was measured to identify differences in care time by patient. Correlations between patient characteristics and care times were calculated. The median time per visit was 49 min and 7 s. Time was mostly spent on daily living assistance (76.0%), followed by medical care assistance (10.0%) and record keeping and administration (14.0%). Results suggested that patient care was characterized by longer time spent on patient comfort, physical therapy, and patient consultation or education. In general, time per visit increased with patients’ age. The variation in care implementation time tended to be large in daily living assistance and small in medical care assistance. These data may be useful for providing and managing personalized care for patients. These data can also contribute to making nurse care more specialized.

## 1. Introduction

Parkinson’s disease (PD) is a progressive neurodegenerative disease that occurs when dopamine-producing nerve cells in the substantia nigra are damaged. In addition to tremor, akinesia, and rigidity, PD symptoms may include dysphagia, excretory disorders, insomnia, and mental health disorders [1,2]. To help patients with this chronic disease comfortably maintain their physical functioning for longer through adequate management of its complicated symptoms, essential care is provided by a nurse with specialized knowledge and skills in PD symptom alleviation and pharmacological and other treatments. Nursing care is also necessary to reduce the stress caused by disease and the burden on caregivers.

In these last 10 to 20 years, interest in PD care and treatment has increased concurrently with a global increase in the number of patients with PD, caused by population aging. In particular, there has been growing interest in care being provided by specialists. In England, PD nurse specialists have been providing patient care for individuals with PD for the last 30 years, and care guidelines have been developed [3,4,5]. Similarly in Japan, the prevalence of PD is rising. Its prevalence rate is 166.8 per 100,000 people [6]. In Japan, PD is designated as an intractable disease. If the patient is in an advanced stage, they are eligible for Public Medical Expenses Subsidy and can receive medical care and nursing care at lower cost when compared to other diseases [7]. According to a national survey conducted in 2016, PD ranked seventh among the causes for needing public Care Services covered by public long-term care insurance [8]. Japan has been warned that it could face a shortage of up to 270,000 nurses by 2025; therefore, a limited number of nursing staff must be able to effectively provide care to those in need [9]. This suggests that it is desirable to set priorities for the type of care the nurse’s time should be used for in the case of patients with PD. To that end, it is necessary to determine what kind of care and how much time is spent on patients with PD, as the information currently available is scarce.

One way to objectively study the type of nursing care being provided to patients with PD is by conducting a time and motion study. These studies have long been conducted to improve the safety and quality of care in the field of hospital healthcare [10]. Several studies have shown that when nurses cared for patients with neuromuscular disease (including PD), most of their time was spent on conversations with the patients and with their family members. Patients with neuromuscular diseases may have had difficulty with speech and may have taken longer to communicate their intentions. In addition, they may have been extremely anxious and would have required a lot of advice. Regarding PD in particular, a considerable amount of time was spent assisting patients with eating, administering medication, and transferring [11,12]. Although these studies suggested the aspects involved in nursing care time for patients with PD characteristically, they did not include a comprehensive view of the nursing care provided. Moreover, these studies have been limited to hospital-based nursing care. Studies in hospitals should differ from studies at homes, in that there are no assistants or family members providing care other than the nurse and there is a nurse present 24 h a day.

Dorsey et al. recommended that patients with PD be moved from treatment facilities to their homes, as this offers key benefits in safety, cost, and patient satisfaction [13]. As progress is made toward developing a system of Community-Based Integrated Care throughout Japan by 2025, the number of patients with PD being treated at home, even if they need medical care, is expected to rise further. Therefore, an increasing number of nurses will be required to effectively provide personalized nursing care for the growing number of patients with PD being treated at home.

A current research gap is the lack of information on the amount of nursing care needed, despite the increasing number of patients with PD and the shortage of nurses. Thus, this study aimed to obtain a comprehensive view of home health nursing, as it is currently being provided to patients with PD, to determine how much variation exists in nursing care time spent with each patient, and to identify patient characteristics that may account for that variation. By clarifying the aspects of home health nursing care time for patients with PD and creating foundational data for future analyses, this study may pave the way for a better understanding of the required home health nursing care, which could be useful for developing a system to provide them with appropriate care in the future.

## 2. Materials and Methods

### 2.1. Participants and Data Collection

From July 2019 through February 2020, a study was conducted with a sample of patients with PD receiving home visits by nurses from a home-visit nursing agency in western Japan. The sample consisted of patients who agreed to participate in the study whose “visiting nursing order” indicated that they had PD. In Japan, home health nurses provide medical care assistance, such as medication and wound care. In addition, they may take care of patients’ meals and toileting. The role of the nurse is the same in the hospital. However, it can be assumed that the amount and content of care in each environment may differ.

Data collected for the study consisted of visiting nursing records and videos taken of the nursing care provided by a nurse during a visit to patients’ homes (or nursing homes). In addition, the study included a questionnaire survey to be completed by the home health nurses to gather data on their characteristics and experience.

#### 2.1.1. The Home Health Nurse Questionnaire

Printed questionnaires were distributed to the nurses visiting the sample patients by the leader of the home-visit nursing agency. Nurses were asked to indicate their age, sex, license data, years of nursing experience, and the number of patients with PD they had cared for in the past.

#### 2.1.2. Visiting Nursing Records of Patients with PD

Data were collected from the face sheet of the patient’s visiting nursing record and the physician’s “visiting nursing order.” These included patients’ age, sex, number of years with PD, stage of disease progression (using the five-stage Hoehn & Yahr scale, hereinafter referred to as “HY stage”—the higher the number, the more advanced the symptoms of PD) [14], household size (including the patient), residence type (home or nursing home), and whether the patient was certified for the Japanese Public Medical Expenses Subsidy for Intractable Diseases.

#### 2.1.3. Video Recording the Nursing Care Provided during a Home Health Visit

According to Lopetegui et al., potential time and motion study methodologies include measurement by external observers, participants’ self-reports, and automated measurements via computerized systems. Additionally, in Japan, unattended time studies (using computerized systems) has become increasingly common [9,15]. This study used 1:1 continuous observation (one nurse observed by one researcher), as recommended by Lopetegui et al., because the study site was at patients’ homes (or room in a nursing home) and only a few participants were involved (the patient and the nurse) [10]. In addition, considering the limited data that were collected, rather than taking notes to record observations, we decided to video record the visit. This was beneficial because multiple observers could verify the categorization of the nursing care activities from the video recording.

To record the videos, a single researcher accompanied the nurse on visits to patients with PD who consented to participate in the study. A home video camera on a tripod was used to record the videos. Based on the assumption that nursing care essential to a patient’s care would be, on average, performed during a single visit, one visit per patient was recorded. Exceptional situations such as emergency visits were avoided. Since the person being filmed was mainly the nurse, inclusion of the patient’s face in the video was avoided. When filming toileting and bathing, the camera angle was changed to avoid including any bare skin in the shot. When that was difficult to avoid, instead of filming, notes were taken on the care provided and the time required to complete said activity.

### 2.2. Ethical Considerations

Written informed consent was obtained from the nurses prior to the study. Permission for a researcher to join the visit was obtained from the patient and their family in advance. Upon arriving at the patient’s place of residence, the researcher asked the patient and the family for their written consent to allow the viewing of the patient’s nursing records and the video recording of the visit. Patients and their families received 3000 yen as an honorarium for their participation. Approval for the study was obtained from the Ethics Committee of the Kobe University Graduate School of Health Science (no. 821).

### 2.3. Analysis

#### 2.3.1. Descriptive Statistics for the Nurses

Data collected on home health care nurses’ characteristics and experience were tabulated.

#### 2.3.2. Descriptive Statistics for Patients with PD

To form a wider perspective of the characteristics of the patients with PD in the sample, data obtained from the visiting nursing records were calculated through descriptive statistics. Spearman’s correlation coefficient was used to determine significant relationships between 4 patient characteristics: age, number of years with PD, HY stage, and household size.

#### 2.3.3. Video-Based Nursing Care Time Analysis

The correlation between each home health nursing care time of patients with PD and patients’ age, sex, number of years with PD, HY stage, household size, and number of visits per month was analyzed by Spearman’s Correlation coefficient.

For the nursing care time analysis, the researchers and the leader of the nursing staff classified the nursing care performed on the video into activity types. If there was ambiguity, the decision was made by checking with the nurse who provided the care. Categories and subcategories were created based on Table A of the Japanese Nursing Association’s New Nursing Service Categories [16]. Nursing care time spent on each activity was measured in seconds. General stopwatches were used to measure time. If two types of activity occurred simultaneously—such as giving the patient advice while giving them a massage—half the time was allocated to each. The entire time of the visit was allocated to one activity or the other. Therefore, tabulating the time spent on nursing care by subcategory produced a comprehensive view of how time was spent during each home health visit.

To visually display variations in time spent on each category and subcategory of nursing care, the data were displayed as box-and-whisker plots. IBM’s SPSS Statistics 27 software (for Windows 10) was used to create the charts and perform the statistical analyses (IBM, Armonk, NY, USA). Significance was set at 10%.

## 3. Results

### 3.1. Descriptive Statistics for the Nurses

Care for the patients with PD was provided by five nurses. All participants were women and had “regular nurse” qualifications (a general nurse registered with the Ministry of Health, Labor, and Welfare of Japan). The mean age for this group was 54.60 ± 10.15 years, the mean years of experience were 32.05 ± 8.23, and the mean for the actual number of patients with PD they had cared for in the past was 65.00 ± 40.42.

### 3.2. Nursing Care Times for Patients with PD

#### 3.2.1. Descriptive Statistics for Patients with PD

Of the 24 patients with PD visited by nurses from this agency, 20 agreed to participate in the study. As shown in Table 1, six (30.0%) of the patients were HY Stage 3, seven (35.0%) were Stage 4, and seven (35.0%) were Stage 5. Eleven patients (55.0%) lived at home and nine (45.0%) lived in nursing homes. These nursing homes are classified as “serviced housing for the elderly” in Japan and are similar to apartment houses in a housing complex. They receive home health nursing and home care services from an outside source for a separate fee. All had been certified as being eligible for the Public Medical Expenses Subsidy for Intractable Diseases. The median age was 78.0 (IQR: 75.3–82.8) years, the median number of years with PD was 13.5 (IQR: 9.3–20.0), the median household size was two (IQR: 1.0–3.0), and the median number of home visits received per month was 9.0 (IQR: 8.0–20.8).

Furthermore, as shown in Table 2, significant positive correlation (*p* = 0.032) was found between number of years with PD and HY stage. In addition, a significant inverse correlation (*p* = 0.032) was found between household size and HY stage.

#### 3.2.2. Total Nursing Care Times

The total time spent during 20 home health visits for the 20 participants was 54,169 s (15 h 2 min and 49 s). Moreover, 15% of that time (8,139 s or 2 h, 15 min, 39 s) was recorded in notes rather than on video because the activities took place during patients’ bathing or toileting. 

The median time spent on a home health visit to each patient was 2,947 s (49 min 7 s). Nursing time spent on patients with PD was a minimum of 1,085 s (18 min 5 s) and a maximum of 3,896 s (1 h 6 min 56 s).

Table 3 shows correlations between the nursing care time of each patient with PD and patients’ characteristics. Nursing care time was positively correlated with patients’ age and household size. A longer total nursing care time was provided to elderly patients (*p* = 0.079). 

#### 3.2.3. A Comprehensive View of Nursing Care Time

Checking the video images with the nurses, nursing care was divided into 15 subcategories. The 15 subcategories were included in three categories: daily living assistance, medical care assistance, and record keeping or administration. Table 4 shows examples of divided nursing care.

**Figure 1:**
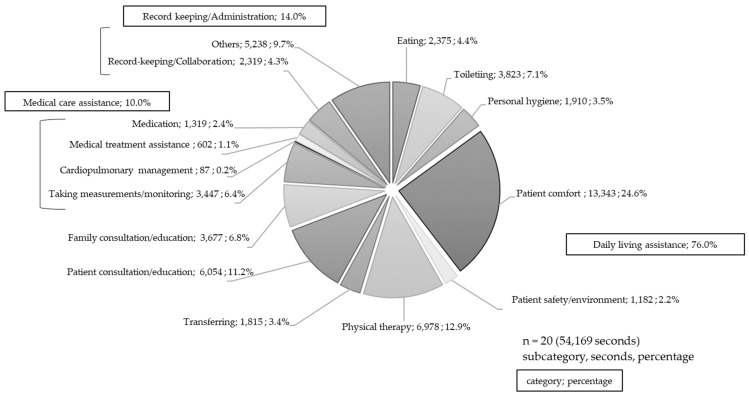
Ration of nursing care times.

Figure 1 shows the ratio of these nursing care times to the total nursing care time. By category, daily living assistance accounted for 76% of nursing care time, medical care assistance accounted for 10.0%, and record keeping or administration accounted for 14.0%. By subcategory, most of the time was spent on patient comfort (24.6%)—which included activities such as massage—followed by physical therapy (12.9%), patient consultation or education (11.2%), toileting (7.1%), family consultation or education (6.8%), and taking measurements or monitoring (6.4%).

#### 3.2.4. Variations in Nursing Care Time Depending on the Patient

**Figure 2:**
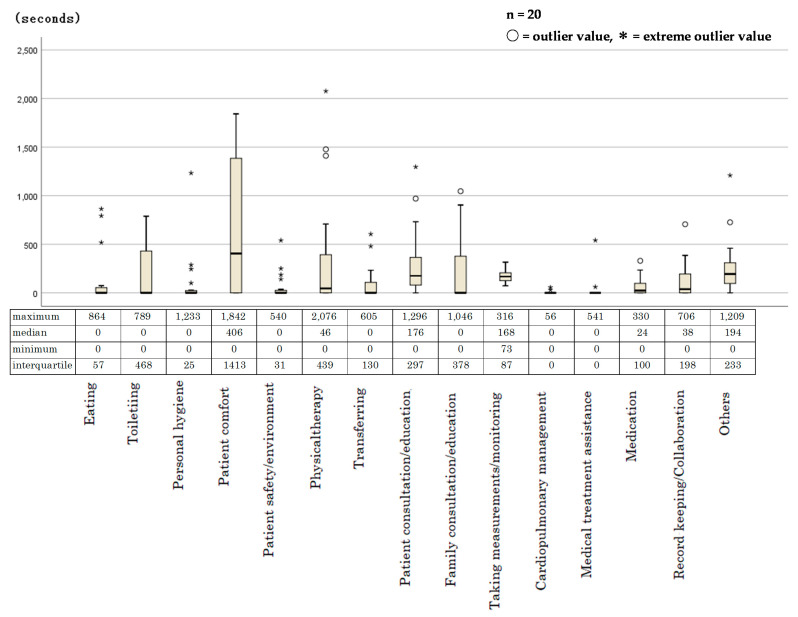
Box and whisker plots for each subcategory.

Figure 2 shows the variation in nursing care time in 15 subcategories using a box-and-whisker plot.

The variation in time spent on the type of care activity is indicated by the width of the boxes (interquartile range) on both sides of the median and the length of the whiskers. The length of the beard is 1.5 times as long as the box. Other than that, it is expressed as an outlier or an extreme outlier. Among the subcategories, those with wider boxes are patient comfort, physical therapy, toileting, family consultation or education, and patient consultation or education. The longer whiskers also indicate a large variation in nursing care time per patient. Extreme outliers were seen in nursing care times for individual patients in the following subcategories: eating, personal hygiene, patient safety or environment, physical therapy, transferring, patient consultation or education, cardiopulmonary management, and medical treatment assistance. The subcategories included in daily living assistance had large variability, whereas those included in medical care assistance had small variability.

## 4. Discussion

### 4.1. Appropriateness of Nursing Care Provided

The averages for age (54.60 years) and years of experience (32.05) for the nurses in this sample were higher than these averages for all nurses in Japan (47.0 and 22.3, respectively) [17]. The fact that, on average, nurses in this study had previously cared for 65 patients with PD highly supported the validity of our assumption that the nursing care they provided to the patients with PD was appropriate. These nursing care practices are empirical assumptions. There are no protocols to follow.

### 4.2. Characteristics of the Patient Sample

All patients with PD in the study were at HY Stage 3 of the disease or higher. This is likely associated with the fact that the need for home health nursing services depended on disease progression. In addition, the costs of these services are covered by Japan’s system of Public Medical Expenses Subsidy Certification for Intractable Diseases for HY Stages 3, 4, and 5.

Therefore, it is possible that patients with HY Stage 3 or above susceptible to nursing care services were present. Furthermore, because all participants were certified as eligible for this subsidy, if the time spent on a visit remained within the 90 min time limit, there were no limitations on the number of visits a patient could receive. Thus, there were no fees for extra services. Therefore, it was important that patients were being provided with all the nursing care time they needed.

The average age among the patient sample was higher than the national and regional average, possibly because participants had progressed to HY Stage 3 or higher [18,19]. Further, the median of nurse visits per month (9.0) was higher than the national average (7.8) [20].

### 4.3. Nursing Care Time Analysis

#### 4.3.1. Relationship between Patient Characteristics and Nursing Care Time

The average time spent on a home health visit for a patient with PD was 49 min 7 s. There was a large difference in nursing care time depending on the patient. According to Table 3, age could have been the patient attribute that contributed the most to the lengthening of time spent on nursing care if one considers the correlations significant at *p* < 0.1. Table 2 shows that there is no significant correlation between age and HY stage, or between age and number of years with PD. This may be because some people have Juvenile Parkinsonism or the age of onset varies from patient to patient. It is interesting to note that nursing care time per visit correlates more strongly with age than with HY stage. The total nursing care time may be influenced by the speed of understanding and the required politeness rather than the speed of patients’ movement. As patients age, the total nursing care time per visit may increase in the future. 

Patients with higher HY stage had significantly smaller household size, suggesting that they may live in nursing homes more often. However, there was no significant association between household size and nursing care time per visit. This may indicate that support other than by home health nurses was provided and that nurses did not over-provide services that could be substituted by others. 

#### 4.3.2. Overall Characteristics of Nursing Care Time

In this study, visiting time was mostly spent assisting patients to improve their daily lives (76.0%). Miyajima reported that, “care for personal belongings”—hygiene, dressing, eating, toileting, transferring, communication, among others—was long also in the hospital [11]. In PD, muscle rigidity and bradykinesia develop because of decreased production of dopamine by cells in the substantia nigra, resulting in motor symptoms in everyday life. In addition, assistance with activities of daily living is frequently required because other non-motor symptoms often develop, such as excretory system problems, sleep disorders, and dementia. In Japan, non-nurses (certified care workers, family members) often provide this care; however, it is necessary to provide high-quality care by nurses because it is necessary to clarify the illness of patients with PD. While this takes a long time, there is a high possibility that it cannot be omitted.

However, in home health nursing, in addition to the aforementioned assistance services to improve patient comfort (such as massage and physical therapy), are frequently provided. Although massage differs from physical therapy in that the patient is only passively involved, it is frequently used with patients complaining of fatigue or pain in their limbs or trunk [4,21]. Thus, a different characteristic of home health nursing from hospital nursing is that a considerable amount of time is devoted to expert massage and physical therapy.

In addition, the amount of time spent on consultation or education for the patients and their families was also relatively long. This highlights the importance of psychological support for the patients and their families and teaching them the skills they need to perform care activities themselves, in addition to providing patients with the nursing care they need. 

#### 4.3.3. Nursing Care Subcategories with Time Variability

According to Figure 2, the large variation in time spent for the patient comfort, toileting, and family consultation or education subcategories might have had a significant effect on total nursing care time. However, time spent assisting patients with medical care was short. The small variation in nursing time needed for this category suggests that the time spent was consistent across all patients. However, a few patients required greater time-intensive care to treat them for pressure ulcers or skin conditions, accounting for significant differences in the time spent.

Although time spent on record keeping and administration was short when compared with medical care assistance, there were higher outliers, suggesting that differences could easily occur depending on the patient. Differences in the time needed for record keeping and collaboration with providers at other institutions depended on a patient’s condition. Similarly, differences in the preparation time likely depended on whether the patient was being tube-fed or receiving other types of treatment or care.

In this study, time measurement was limited to the time between the beginning and end of a nurse’s visit to a patient’s home (or nursing home room); thus, the time spent on record keeping and administration was limited to the visit duration. Therefore, one feature of this analysis was that nursing care time that would have taken place in an office (e.g., phone calls, conferences, record keeping, and preparation) was not considered. Nagata et al. found that when measuring total nursing care time per month, intractable diseases (e.g., neuromuscular disease) were the most time-intensive, with a significant amount of time being spent on collaboration with other institutions and in conferences [22]. Although a separate study is needed to examine nursing care time spent outside of patients’ homes, this study suggests that a few patients’ needs demand substantial time be spent on record keeping and administration.

#### 4.3.4. Nursing Care Time Management

The fact that home health nursing care for patients with PD requires considerable care time for patient comfort, physical therapy, patient consultation or education, toileting, and family consultation or education is important. What is notable is the fact that these nursing cares cause variability in care time. The variability is caused by the needs of each patient with PD and should be perceived positively. Nurses need to accurately assess the care needed for each patient and enhance the implementation of the care needed. Nurses should always consider personalized care for patients with PD and manage their time. These data can also contribute to making nurse care more specialized and sophisticated.

### 4.4. Study Limitations

The limitations of this study are that it is limited to one agency and only measured nursing time for one visit for twenty patients. Nursing time per visit was negatively correlated with the number of visits per month. Patients who are visited more often during the month may have shorter nursing hours per visit. More data need to be collected to analyze the total amount of nursing time required for patients with PD.

Furthermore, to properly generalize these results, it would also be necessary to remove the impact of patient comorbidities (other than PD) on nursing care time.

## 5. Conclusions

The median time spent per home health visit to 20 patients with PD was 49 min 7 s and elderly patients had longer nursing care times per treatment (*p* < 0.1). Time may increase as patients age.

Much of that nursing care time was spent on patient comfort, physical therapy, patient consultation or education, toileting, and family consultation or education. This suggests that these are the characteristics of home health nursing, which supports independently living among patients with PD by alleviating symptoms and helping them maintain their physical functioning. 

The care described above caused variations in nursing care time. It is necessary for nurses to perform time management, understanding that nursing care will be longer for patients who require said care.

## Figures and Tables

**Table 1:** Descriptive statistics for patients with Parkinson’s disease.

	*n* = 20	
	Breakdown	(%)
Male	10	(50.0%)
Female	10	(50.0%)
HY Stage 1	0	(0.0%)
HY Stage 2	0	(0.0%)
HY Stage 3	6	(30.0%)
HY Stage 4	7	(35.0%)
HY Stage 5	7	(35.0%)
Lives in own home	11	(55.0%)
Lives in nursing home	9	(45.0%)
Registered for Public Medical Expenses Subsidy Certificate	20	(100.0%)
Not registered for Public Medical Expenses Subsidy Certificate	0	(0.0%)
	Median	(IOQ)
Age (i.e., years)	78.0	(75.3–82.8)
Number of years with PD	13.5	(9.3–20.0)
Household size (including self)	2.0	(1.0–3.0)
Number of visits per month	9.0	(8.0–20.8)

HY Stage 1: PD symptoms on one side of the body, HY Stage 2: PD symptoms on both sides of the body, HY Stage 3: Mild to moderate PD symptoms, postural instability and need assistance in daily activities, HY Stage 4: Shows severe disability, but somehow walks possible without assistance, HY Stage 5: Living in bed or in a wheelchair without assistance.

**Table 2:** Correlations between 4 patient characteristics.

		Age (i.e., Years)	Number of Years with PD	HY Stage	Household Size (Including Self)
		cc	cc	cc	cc
		*p*	*p*	*p*	*p*
Age (i.e., years)	cc	1.000	−0.120	0.223	−0.127
	*p*	-	0.614	0.344	0.595
Number of years with PD	cc	−0.120	1.000	0.479	−0.368
	*p*	0.614	-	0.032 *	0.111
HY stage	cc	0.223	0.479	1.000	−0.480
	*p*	0.344	0.032 *	-	0.032 *
Household size (including self)	cc	−0.127	−0.368	−0.480	1.000
	*p*	0.595	0.111	0.032 *	-

† <0.1, * *p* < 0.05, ** *p* < 0.01; cc = Spearman correlation coefficient; cc. and *p*-values have been rounded off; 4 patient characteristics did not show normal distribution by Kolmogorov-Smirnov’s normality test.

**Table 3:** Correlations between nursing care time and patient characteristics.

		Age (i.e., Years)	Number of Years with PD	HY Stage	Household Size (Including Self)	Number of Visits per Month
Total time spent on a home health visit (per person)	cc	0.402	−0.327	−0.069	0.330	−0.294
	*p*	0.079 †	0.160	0.771	0.155	0.209

† *p* < 0.1; cc = Spearman correlation coefficient; cc and *p*-values have been rounded off.

**Table 4:** Examples of divided nursing care.

Category	Subcategory	Nursing Care (Example)
Daily living assistance	Eating	Tube feeding, Water intake, Oral intake
	Toileting	Indwelling bladder catheter, Stool extraction/enema, Abdominal massage, Urine withdrawal, Rectal colostomy, Genital cleaning, Movement to the restroom, Diaper changing
	Personal hygiene	Bathing, Changing clothes, Personal grooming, Oral care
	Patient comfort	Massage/passive limb exercise, Posture/positioning
	Patient safety/environment	Setting of room temperature and illumination, Clean up and organize the room, Fixation of safety fence and confirmation of bed height
	Physical therapy	Swallowing/speaking exercise, Active limb exercise
	Transferring	Wheelchair movement assistance, Walking movement assistance
	Patient consultation/education	Patient consultation/education
	Family consultation/education	Family consultation/education
Medical care assistance	Taking measurements/monitoring	Measurement of vital signs, Monitoring
	Cardiopulmonary management	Aspiration (oral), Helping expel phlegm, Cardiopulmonary sound auscultation
	Medical treatment assistance	Bedsore treatment, Other skin treatments
	Medication	Classification and checking of medication, Dosage, application of topical medication, Dosage through a feeding tube
Record keeping or administration	Record-keeping/Collaboration	Filling out collaboration notebooks, Filling out nursing records
	Others	Preparation/clean-up, Waiting, Confirmation of monthly medical expense subsidy amounts

## Data Availability

The data used to support the findings of this study are available from the corresponding author upon request.
